# Does the National Credit Demonstration Policy Affect Urban Green Economy Efficiency? Evidence from the Yangtze River Delta Region of China

**DOI:** 10.3390/ijerph19169926

**Published:** 2022-08-11

**Authors:** Haisheng Chen, Dingqing Ni, Shuiping Zhu, Ying Ying, Manhong Shen

**Affiliations:** 1 College of Economics and Management, Zhejiang A&F University, Hangzhou 311300, China; 2 Institute of Ecological Civilization, Zhejiang A&F University, Hangzhou 311300, China; 3 Research Academy for Rural Revitalization of Zhejiang Province, Zhejiang A&F University, Hangzhou 311300, China; 4 Zhejiang Provincial Credit Center, Hangzhou 310007, China; 5 Wenzhou Pharmaceutical Industry Development Co., Wenzhou 325099, China; 6 College of Environmental Science and Engineering, Donghua University, Shanghai 201620, China

**Keywords:** national credit demonstration policy, green economy efficiency, regional heterogeneity, institutional supply, distortionary effects

## Abstract

A more scientific green economy efficiency indicator is constructed based on OH (2010), and a multiperiod spatial DID model is used to examine the impact of national credit demonstration policies on urban green economy efficiency in a sample of cities above the prefecture level in the Yangtze River Delta. The study confirms the following: (1) The national credit demonstration policy makes a significant contribution to the green economic efficiency of cities, and it is conducive to strengthening awareness of the rule of law in the market to regulate market order. (2) The demand for credit regulation in coastal areas has increased under the new development pattern, and the national credit demonstration policy has effectively enhanced green economy efficiency through institutional supply. (3) Under the national credit demonstration policy, the subprovincial level and above can mobilise more resources for policy refinement and support, reducing transaction costs and improving the efficiency of the green economy. (4) The impact of the national credit demonstration policy on the efficiency of Zhejiang’s green economy is more obvious; but, under the overall framework of the Yangtze River Delta, the policy has a more prominent role in promoting green economy efficiency in other provinces. Policy insights are as follows: (1) Different cities have different degrees of impact on the efficiency of the green economy from the national credit demonstration policy, and they should implement differentiated measures based on regional heterogeneity; (2) regulating the use of administrative resources and avoiding undue administrative intervention are important prerequisites for promoting regional integration to enhance the efficiency of the green economy; and (3) strengthening interprovincial credit policy synergies can help to alleviate administrative distortions of policy implementation and enhance the efficiency of the regional green economy.

## 1. Introduction

The concept of “green development” was first introduced by the United Nations Development Programme in 2002, and it is widely regarded as the ideal path to achieving organic integration of the economy and the environment [1]. In May 2008, the Chinese government introduced regulations on the disclosure of pollution data from government departments, with a focus on promoting green and sustainable economic development. A central question asked by this study is: What is the level of efficiency of China’s green economy, as represented by the Yangtze River Delta region, under the current environmental regulations? Given the accelerated advancement of the social credit system, it is of great theoretical and practical value to examine the impact of the national credit model policy on the efficiency of the green economy at the city level, to grasp the practical effects of green economic development under environmental credit regulation, and to formulate and improve public policies on environmental credits in China in the next phase.

To enhance the quality of urban green economic development, China has introduced and implemented credit demonstration policies since 2015; by 2019, a total of 43 cities (urban areas) in two batches were approved as national demonstration cities for the creation of social credit systems. The Yangtze River Delta region is one of the regions with the most intensive implementation of national credit demonstration policies, with 14 cities (urban areas) having been approved as national credit demonstration pilot areas. As an adaptive institutional innovation, the National Credit Demonstration Policy can be applied to both command-and-control environmental regulation models and transformed into a self-fulfilling model of green governance based on market cost–benefit trade-offs, which emphasises the use of market instruments, such as green credits and green investment with social instruments, such as information disclosure and public participation, as well as the integration of the roles of the three mechanisms—government, market, and society—in practice. This is conducive to promoting the diversification of instruments for green economy governance. At the same time, the national credit model policy is in line with the general value recognition of the public, and it has gained the implicit consensus of society, which is of great practical significance in promoting the interface between the formal system of regulations and policies and the informal system of public green expectations.

In fact, national credit demonstration policies may have heterogeneous effects on the efficiency of urban green economies. On the one hand, national credit demonstration policies may be more conducive to the development of environmentally friendly enterprises and the efficiency of urban green economies by optimising and enhancing the business environment. Firstly, by seeking to be included in the environmental credit red list, green enterprises may gain a better advantage in terms of market access, access to resources, project support, and regulatory flexibility, which will have a positive impact on their green production efficiency. Secondly, under the reputation mechanism, companies with a good environmental label are more likely to be recognised by their associates and the public in the market and can better address the financing constraints they face through the commercial credit route. Finally, in China’s particular context, green enterprises are encouraged more by the government, and those in charge of them gain political affiliation by seeking positions as deputies to the National People’s Congress and members of the Chinese People’s Political Consultative Conference in order to better understand development policies, reorient their businesses, and improve their production capacity, which has the potential to increase the efficiency of the green economy at the regional level. On the other hand, the strengthening of the social credit system in cities under the national credit model policy may lead some polluting enterprises to relocate across regions for fear of being blacklisted and subject to joint interdepartmental disciplinary action, which could lead to inter-regional changes in green economy efficiency. In the absence of national social credit regulations, the degree of environmental credit regulation varies from province to province, and under the “pollution sanctuary” effect, some enterprises may choose to locate in provinces with less stringent environmental regulations on balance, which will have a profound impact on the efficiency of the green economy inside and outside of the region, given the tendency of industries to cluster. This will have a profound impact on the efficiency of the green economy in both inbound and outbound regions. Therefore, using the Yangtze River Delta region as a research case, the article examines the possible heterogeneous effects of national credit demonstration policies on urban green economic efficiency on the basis of a reasonable measurement of urban green total factor productivity. Considering the assessment of policy effects involving the pilot social credit system, a double difference approach (DID) [2] is used to analyse the impact of the national credit demonstration policy on the green economic efficiency of Yangtze River Delta cities.

## 2. Literature Review

The literature on the impact of national credit demonstration policies on the efficiency of urban green economies is sparse and develops along three main paths. First is the information-based effects of green credit regulation. The reality is that information is asymmetric, and if it is symmetric then many institutions are equivalent. Information costs are the main determinant of institutional effectiveness, but the Grossman–Stiglitz paradox reveals that, in reality, information asymmetry is the norm [3], so different regulatory regimes correspond to different efficiencies. Information-based credit regulation tools leverage reputation effect to impose green constraints on micro-enterprise production behaviour, including not only government-mandated disclosure of environmental credit information generated in the course of an enterprise’s operations but also the voluntary external release of information about the enterprise’s commitment to green social responsibility. Given that the right to know about the environment is an important prerequisite for citizens to reasonably exercise their environmental rights, the disclosure of necessary environmental credit information by government and enterprises is also a manifestation of fulfilling social obligations. With a focus on integration and intelligence, the social credit system provides panoramic, full-chain, digital credit supervision and services by collecting and publicising corporate green credit data and integrating the core business of national, provincial, municipal, and county environmental departments’ credit, which is an extension of the existing green information disclosure system. As the green credit situation is an important dimension affecting the reputation of enterprises, the construction of a social credit system can play a subtle role in guiding corporate behaviour and even green economic efficiency.

The second path is the market-based effect of green credit regulation. A network of relational contracts between individuals, businesses, political groups, and organisations is typical of a market economy. For relational contracts to be successful, formal and informal credit regulation and enforcement systems are essential. Market-based green regulation tools replace traditional command-and-control regulation with more flexible incentive-based regulation, covering not only green taxes, green subsidies, and green procurement, but also green financial policies, such as green credit, green bonds, and green investment, which are promoted by the government in conjunction with financial institutions. Through the docking of financial comprehensive service platforms at all levels with the national credit and credit platform, the sharing and integration of public credit data with socialised data such as financing and credit, market transactions, etc. is promoted, and the situation of enterprises’ green behaviour is incorporated into the decision-making framework of public resource allocation and financial credit, which strongly promotes the quality of green economic development in the city. In addition, the classification, stratification, and subsystem collaborative supervision of enterprises with different green credit status is a useful complement to information-based green regulation tools, expanding the breadth and depth of the application of social credit systems in the market sector and helping to enhance the efficiency of the regional green economy.

The third path is the green credit regulation effect that stimulates self-implementation. An effective system must be self-fulfilling. In the process of incentivising self-implementation, the social credit system makes information on reputations more public through the establishment of appropriate punishment and incentive mechanisms, raising the incentive for the green development of enterprises and guiding them to comply with the green credit system, carry out green credit commitments that are compatible with the legal requirements, undertake higher levels of responsibility for green development, consciously fulfil their environmental obligations to pursue green production, and thus improve urban green economic efficiency. Therefore, the incentive for self-implementation of green regulation is not only an important prerequisite for improving the construction of the social credit system but also an important practice for strengthening the integration of environmental credit regulation with “moral governance” and “good governance” and for improving the quality of regional green economic development [4].

In fact, as an important pilot policy to promote the construction of China’s social credit system, the implementation content and assessment indicators of the National Credit Demonstration Policy are generally consistent with the guidelines and directions of the construction of the social credit system. However, the existing studies are somewhat one-sided, either analysing the implementation path, interprovincial spatial interaction, and possible impact of the national credit demonstration policy on green development efficiency from a macro level or studying the possible alleviation effect of the national credit demonstration policy on enterprise financing constraints at the micro level. We sought to examine the effect of credit demonstration policies on the efficiency of the green economy.

## 3. Indicator Construction, Data Description, and Measurement Model Setting

### 3.1. Indicator Construction

#### 3.1.1. Green Economic Efficiency

The exclusion of resource factors does not fully reflect the characteristics of economic development. Therefore, some scholars have incorporated resource and environmental factors into productivity measurement models and used different methods to measure green economic efficiency [5,6,7,8,9,10,11,12,13]. For example, Chung et al. (1997) [14], in their work on measuring total factor productivity (TFP) in Swedish pulp mills, for the first time included pollution emissions as an undesired output and established a directional distance function (SBM). However, with the SBM model it is difficult to solve the problem of co-existence of radial and nonradial methods, so Tone [15] innovated a hybrid distance function EBM model, which incorporates slack variables into the function and considers both a CCR model [16] with radial factors and a nonradial SBM model with slack variables, which weakens the measurement error that may arise from a single distance function. This improves the accuracy and the realistic applicability of the model for measuring green technological progress.

Kuosmanen [17] combined the strengths of the DEA and SFA models to construct a stochastic semiparametric data envelope model to analyse agricultural green productivity across countries for the period 1990–2004. Zhang Yanan et al. (2022) measured the green total factor productivity of the Huaihe Economic Zone from 2004 to 2017 based on the carbon cycle, and used the spatial Durbin model to analyse the effects of seven variables on green total factor productivity, including the level of economic development, environmental regulation, the level of R&D, and the degree of openness to the outside world [18]. Yuanxin Peng et al. (2022) used the Malmquist index, spatial autocorrelation analysis, and convergence analysis to analyse GTFP in 263 prefecture-level and above cities in China [19]. Zhu Yingyu et al. (2022) measured green total factor productivity based on the net carbon sink in China’s plantation sector using a stochastic frontier analysis with output-oriented distance function based on panel data from 30 Chinese firms from 2001 to 2019, and empirically examined the impact of agricultural mechanisation on green total factor productivity [20]. Based on provincial panel data from 2008 to 2017, Yining Zhang et al. (2022) measured the green total factor productivity of China’s manufacturing industry using the Malmquist–Luenberger (ML) model and further constructed an empirical model to analyse the influence mechanism of green total factor productivity [21]. Chen Haisheng et al. (2022) used the Slack-Based Model (SBM) Global Malmquist–Luenberger (GML) index to measure green total factor productivity in China’s agriculture by province and used social network analysis (SNA) and vector autoregressive model (VAR) impulse response function (IRF) to examine the green total. The spatial network structure and regional interactivity of green total factor productivity in China were investigated using social network analysis (SNA) and a vector autoregressive model (VAR) impulse response function (IRF) [22].

Following Oh’s [23] research, the results of the GML index measure of the SBM model were used to characterise the green economic efficiency of the city. In this case, the mathematical expression of the SBM model is:

(1) $$\rho^{*}=\min\frac{\frac{1}{m}\sum_{i=1}^{m}\frac{{\bar{x}}_{i}}{x_{i0}}}{\frac{1}{S_{1}+S_{2}}(\sum_{r=1}^{S_{1}}\frac{{\bar{y}}_{r}^{g}}{y_{r0}^{g}}+\sum_{r=1}^{S_{2}}\frac{{\bar{y}}_{r}^{b}}{y_{r0}^{b}})},s.t.\{\begin{array}{c} \bar{x}\geq \sum_{j=1,\neq k}^{n}\theta_{j}x_{j}\\ {\bar{y}}^{g}\leq \sum_{j=1,\neq k}^{n}\theta_{j}y_{j}^{g}\\ {\bar{y}}^{b}\geq \sum_{j=1,\neq k}^{n}\theta_{j}y_{j}^{b}\\ \bar{x}\geq x_{0},{\bar{y}}^{g}\leq y_{0}^{g},{\bar{y}}^{b}\geq y_{0}^{b},{\bar{y}}^{g}\geq 0,\theta \geq 0 \end{array}$$

$$x\in R^{m},y^{g}\in R^{S_{1}},yb\in R^{S_{2}}$$

$$X=[x_{1},x_{2},\ldots ,x_{n}]\in R^{m\times n},Y^{g}=[y_{1}^{g},y_{2}^{g},\ldots ,y_{n}^{g}]\in R^{S_{1}\times n},Y^{b}=[y_{1}^{b},y_{2}^{b},\ldots ,y_{n}^{b}]\in R^{S_{2}\times n}.$$

The SBM model is based on the assumption of constant size; $S=(S^{-},S^{g},S^{b})$ represents the input, desired, and undesired output slack; and the objective function value ρ* characterises the efficiency value of the decision unit.

The mathematical expression for the GML index is:

(2) $$\begin{array}{c} GML^{t,t+1}=\frac{1+{\vec{D}}_{o}^{G}(x^{t},y^{t},b^{t};y^{t},b^{t})}{1+{\vec{D}}_{o}^{G}(x^{t+1},y^{t+1},b^{t+1};y^{t+1}b^{t+1})}\\ =\frac{1+{\vec{D}}_{o}^{G}(x^{t},y^{t},b^{t};y^{t},b^{t})}{1+{\vec{D}}_{o}^{G}(x^{t+1},y^{t+1},b^{t+1};y^{t+1}b^{t+1})}\times \frac{1+{\vec{D}}_{o}^{G}(x^{t},y^{t},b^{t};y^{t},b^{t})/1+{\vec{D}}_{o}^{G}(x^{t},y^{t},b^{t};y^{t},b^{t})}{1+{\vec{D}}_{o}^{G}(x^{t+1},y^{t+1},b^{t+1};y^{t+1}b^{t+1})/1+{\vec{D}}_{o}^{G}(x^{t+1},y^{t+1},b^{t+1};y^{t+1}b^{t+1})}\\ =EC^{t,t+1}\times TC^{t,t+1}. \end{array}$$

The *GML* index can be decomposed into technical efficiency (*EC*) and technical progress (*TC*) [24], where *x*, *y*, *b*, and *t* denote the input, desired output, undesired output, and time, respectively. ${\vec{D}}_{o}^{G}(x^{t},y^{t},b^{t};y^{t},b^{t})$ and ${\vec{D}}_{o}^{G}(x^{t+1},y^{t+1},b^{t+1};y^{t+1},b^{t+1})$ denote the efficiency values of the decision unit in period *t* and period *t* + 1, respectively.

**Figure 1:**
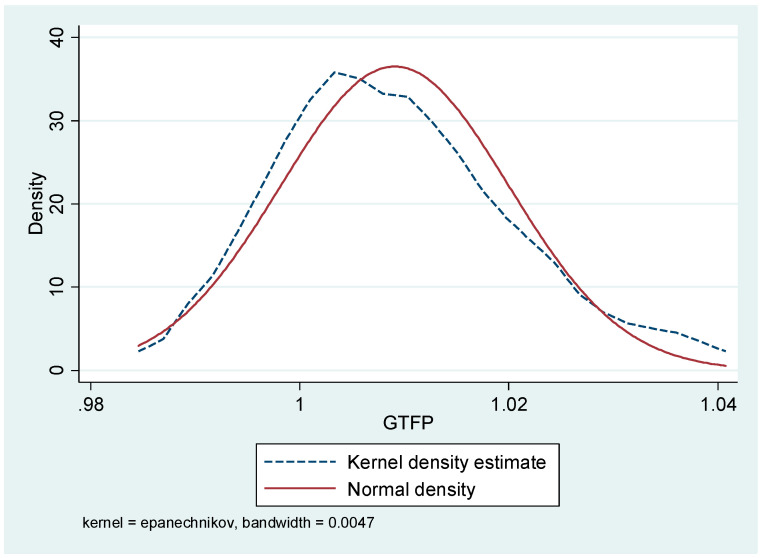
Kernel density diagram of urban green economic efficiency measurement results.

In the measurement of urban green economy efficiency, the selection of input indicators includes the following: (1) Capital input. The physical capital stock of the city is selected for measurement. Due to the lack of corresponding statistical indicators, this paper uses the method of Shan Howie (2008), using the data of fixed asset investment flow in each city, and the base period of 2010 for deflating in which the depreciation rate is set at 10.96%. (2) Labour input. The total number of employees in secondary and tertiary industries was selected as the indicator of labour force in a particular city. (3) Resource and energy inputs. Total water supply and social electricity consumption were chosen as the indicators for measuring resource and energy inputs in the economic development of the city, respectively. (4) Desired output. The level of economic development and the quality of life of urban residents are considered as the main indicators of desired output, and the real gross regional product and greening coverage of built-up areas are selected as proxy variables for the above two indicators, respectively. (5) Undesired output. We focused on the selection of industrial smoke emissions, industrial wastewater emissions, industrial SO_2_ emissions, and PM2.5 concentrations as indicators to measure the pollution situation in the process of urban economic development. From the kernel density plot [25] (Figure 1), although the measured green economic efficiency of the Yangtze River Delta cities shows a right-skewed distribution overall, the difference from the normal distribution is not obvious, and the measurement results generally meet the statistical requirements.

#### 3.1.2. National Credit Demonstration Policy

In August 2015, the National Development and Reform Commission (NDRC) and the People’s Bank of China (PBOC) jointly issued a document to implement the national credit demonstration policy, emphasising the in-depth promotion of the social credit system construction through policy innovation, specifying the cities of Nanjing, Wuxi, Suqian, Hangzhou, Wenzhou, Yiwu, Hefei, and Wuhu in the Yangtze River Delta region as the first batch of cities to create a national demonstration city for social credit system construction. In April 2016, the NDRC and the People’s Bank of China approved the work plan of 32 cities to create national credit demonstration cities, and Shanghai Pudong New Area and Jiading District in the Yangtze River Delta, Suzhou City in Jiangsu, Taizhou City in Zhejiang, and Anqing City and Huai Bei City in Anhui were listed as the second batch of urban areas to create national demonstration cities for the construction of a social credit system. Considering that the study was conducted on cities above the prefecture level, Yiwu was included in Jinhua, and Shanghai Pudong New Area and Jiading District under Shanghai for analysis. Therefore, the eight cities in the Yangtze River Delta region that were approved as the first batch of national credit demonstration creation in 2015 and the five cities that were approved as the second batch of national credit demonstration creation in 2016 were used as the treatment group for the multiperiod double-difference model in this paper, while the other 28 cities were used as the study control group.The urban characteristics of the Yangtze River Delta region are analysed in Table 1.

#### 3.1.3. Control Variables

Following the research of Taskin and Zaim [26] et al., control variables such as ownership structure (X1), level of economic development (X2), industrial structure (X3), degree of openness to the outside world (X4), degree of government intervention (X5), advanced degree of labour market (X6), level of technological innovation (X7), and degree of environmental regulation (X8) were selected for inclusion in the model for econometric estimation. According to the new institutional economics and the environmental Kuznets curve, the ownership structure and level of economic development are important factors affecting the efficiency of urban green economies. Government intervention and the degree of environmental regulation have an impact on green total factor productivity under the goal of peak carbon neutral policies. The “pollution sanctuary” hypothesis implies that the external linkages and industrial structure characteristics of a region cannot be ignored when talking about green development. The level of technological innovation plays a role in the way a region develops and sustains a green economy. In addition, labour market conditions, which involve the potential for division of labour, may also have an impact on the efficiency of urban green economies.

The ownership structure (X1) is measured by the share of private and self-employed workers in the total number of employees; the level of economic development (X2) is characterised by GDP per capita; and the industrial structure (X3), the degree of openness to the outside world (X4), the degree of government intervention (X5), and the level of scientific and technological innovation (X7) are measured by the value added of the secondary industry, the amount of foreign capital actually used in the year, the general budget expenditure of local finance, and the share of R&D internal expenditure in the regional GDP, respectively. R&D internal expenditure is a proportion of regional GDP, the degree of advanced labour market (X6) is measured by the number of university students per 10,000 people in cities, and the degree of environmental regulation (X8) is measured by the centralised treatment rate of urban sewage treatment plants. In summary, the inclusion of institutions, factors, and the environment as control variables can mitigate the problem of missing explanatory variables.

### 3.2. Description of Data

Considering the research needs and data availability, the analysis targets 41 cities above prefecture level and three provinces in the Yangtze River Delta, and the research interval is 2010 to 2019. The input and output data were obtained from the *China City Statistical Yearbook*, the statistical bulletins of each province and city and the EPS data platform. The PM2.5 concentration data of different cities were obtained from the satellite remote sensing data published by the National Aeronautics and Space Administration (NASA), and the 1:4 million Chinese basic geographic information data provided by the National Basic Geographic Information Centre (NBIC) were cropped to obtain the average PM2.5 concentration values of the cities for each year. For missing data, the interpolation method was used to process the data.

### 3.3. Measurement Models

In examining the impact of the National Credit Demonstration Policy on urban green economy efficiency, the traditional multiperiod DID model has the advantage of analysing the effect of policy implementation, with cities belonging to the treatment group when they are affected by policy implementation and the control group when they are not affected by policy implementation; at the same time, a time dummy variable for policy implementation is introduced, with the year before the policy implementation taking a value of 0 and the year after the policy implementation taking a value of 1. The basic model formulation is as follows:

(3) $$GTFP_{it}=\beta_{0}+\sum_{k=1}^{K}X_{it,k}\beta_{k}+DID_{it}\beta_{k+1}+\varepsilon_{it}$$

where *GTFP* represents the level of green economy efficiency and *β* represents the regression coefficient of each variable; *t* represents the time of implementation of the National Credit Demonstration Policy and takes values within [1, T], T = 10. *X_it__,__k_* represents the k control variables in the model and takes values within [1, K], K = 8. *DIDit* represents the dummy variable interaction term, which is the policy effect parameter to be estimated, and is obtained by multiplying the values of the group attributes and the dummy variables at the point of approval of the National Credit Demonstration Policy and by centralisation. The dummy variable values at the point of policy approval are multiplied together and centralised. *εit* represents the random error.

However, the magnitude of the effect of the national credit demonstration policy on the green economic efficiency of cities in the context of the construction of a large national unified market gradually weakens, and the green development of cities is increasingly influenced by neighbouring cities, especially in the context of the integrated development of the Yangtze River Delta arising as a national strategy. The spatial interaction of the national credit demonstration policy in the implementation process will also be more obvious. In 2019, the global univariate Moran indices for the interaction term and green economic efficiency were −0.1442 and −0.1511, respectively, and the global bivariate Moran index for the interaction term and green economic efficiency was −0.1222 (Table 2), confirming that there is a relatively obvious spatial correlation between urban green economic efficiency and the effect of national credit demonstration policies on the implementation of green economic efficiency [27], and therefore a spatial econometric model is used in parallel with the traditional OLS model to carry out the corresponding analysis, taking spatial correlation into account. According to the different impacts of spatial correlation, it can be further subdivided into a spatial lag model (SLM) and a spatial error model (SEM). Compared to SLM, SEM is special in that the spatial dependence effect is present in the error term and measures the extent to which shocks to the surrounding area regarding the error of the dependent variable act on the observations in the region.

In light of the above discussion, a more scientific multiperiod spatial DID model is constructed on the basis of the traditional multiperiod DID model, taking into account the requirement of spatial multicollinearity avoidance, to provide a targeted analysis of the impact of national credit demonstration policies on urban green economic efficiency. In particular, the spatial lagged model (SLM) is formulated as follows:

(4) $$GTFP_{it}=\sum_{it=1}^{NT}\rho_{SLM}{\left(\xi ⊗\pi \right)}_{it,it}GTFP_{it}+\beta_{0}+\sum_{k=1}^{K}X_{it,k}\beta_{k}+DID_{it}\beta_{k+1}+\varepsilon_{it}$$

The spatial error model (SEM) is formulated as follows:

(5) $$GTFP_{it}=\beta_{0}+\sum_{k=1}^{K}X_{it,k}\beta_{k}+DID_{it}\beta_{k+1}+\mu_{it}$$

(6) $$\mu_{it}=\sum_{it=1}^{NT}\rho_{SEM}{\left(\xi ⊗\pi \right)}_{it,it}\mu_{it}+\varepsilon_{it}$$

where *ξ* and *π* denote the temporal and spatial weight matrices after row normalization, respectively. ξ⊗π represents the endogenous spatiotemporal weight matrix, which is measured according to the city location relationship: the matrix element is 1 if the two cities are adjacent in spatial location and 0 otherwise. In addition, *i* = 1, 2,…, *N*, where *N* = 41, denoting the 41 cities above prefecture level in the Yangtze River Delta region; *ρ* represents the spatial correlation coefficient in the spatial econometric model, and μ represents the normally distributed random error vector.

## 4. Estimation of Measurement Results

### 4.1. Baseline Model Estimation Results

Based on spatial correlation tests of the core variables, a multiperiod spatial DID approach was used to test the impact of national credit demonstration policies on urban green economic efficiency. The benchmark model was carried out using stepwise regression and the estimation results are reported in Table 3, where OLS, SLM, and SEM denote the ordinary least squares model, spatial lag model, and spatial error model, respectively. In the next step, we chose the most appropriate model of the three (OLS, SLM, or SEM) to carry out the discussion based on the judgement rules. Combining the Log-likelihood, AIC, and SC value magnitudes, Models (3), (6), (9), and (12) were selected for focused analysis. With the number of control variables being 5, 7, and 8 respectively, the treatment effect coefficients of national credit demonstration policies on urban green economic efficiency were 0.020, 0.027, and 0.026, and they were significant at the 10%, 5%, and 5% levels, indicating that national credit demonstration policies had a strong positive correlation with urban green economic efficiency, and cities with national credit demonstration policies had generally higher economic efficiency levels. The economic explanation is that the overall green economic efficiency of a region is closely related to the degree of market development in that region, and as an innovative means to promote the construction of a social credit system, the National Credit Demonstration Policy actively explores the in-depth application of social credit in the areas of government, the market, and society, emphasising both the use of market instruments, such as green credit and green investment, and social instruments, such as information disclosure and public participation, and the integration of the roles of government, market, and society. This is of great practical significance for strengthening awareness of the rule of law in society, regulating the order of socialist market development, and enhancing the efficiency of the urban green economy.

Of the control variables, regional industrial structure (X3) was positively correlated with urban green economy efficiency, with the regression coefficient passing the significance test at the 5% level. This indicates that the optimisation of the industrial structure is of great practical significance for reducing pollution emissions, improving resource utilisation efficiency, and promoting high-quality development of the green economy. The degree of government intervention (X5) had a positive effect on the efficiency of the urban green economy. Under the background of the normalised prevention and control of the new crown epidemic, the implementation of active fiscal policy and the promotion of an effective market through government action are conducive to stabilising market expectations, expanding market demand, and improving the efficiency of green economic development. The effect of the level of science and technology innovation (X7) on the city’s green economic efficiency is negative (passing the 5% significance test), which shows some deviation from expectations. One possible explanation is that the effect of R&D expenditure often has a certain lag, and the economic benefits of current research investment are difficult to show directly in the short term, and must be gradually presented in a larger time dimension. The economic benefits of current research investments are often not directly visible in the short term.

### 4.2. National Credit Demonstration Policies and Green Economy Efficiency: Locational Variability

The initial endowments and evolutionary paths of regional economic development are different. The early advantages of the Yangtze River Delta coastal region (Shanghai, Hangzhou, Ningbo, and other cities) during the reform and opening-up have gradually widened the gap in economic development between it and noncoastal regions (Suqian, Lishui, Huangshan, and other cities) due to the “path-dependent” effect. On the whole, the market economy in the coastal areas is characterised by a better functioning mechanism and a higher quality of the public and commercial credit environment. On the one hand, in a modern market economy, the concepts of rule of law and credit are deeply rooted, especially in the context of building common wealth, and corporate reputation assumes an increasingly important role in areas such as access to resource factors, competition for soft power in development, and enhancement of brand premium capacity. On the other hand, compared with noncoastal regions, coastal regions have a more standardised social credit system and green regulations, and are in a leading position in the competition for regional comparative advantages, which plays a leading role in the improvement of green credit systems in other regions. Therefore, an interaction term between coastal regions, noncoastal regions, and national credit demonstration policies was introduced into the benchmark model, and the regression results on the impact of national credit demonstration policies on green economic efficiency are reported in Table 4.

Combining the Log-likelihood, AIC, and SC value magnitudes, models (5) and (9) were selected for the next step of the analysis. It was found that the effect of the national credit demonstration policy on urban green economic efficiency had significant locational variability, with the effect of the national credit demonstration policy on green economic efficiency in coastal areas being significantly positive at the 5% statistical level and the coefficient of the interaction term x (coastal, noncoastal), the treatment effect coefficient, being 0.043, while this effect was not evident in noncoastal areas. The economic explanation is that, at this stage, the Yangtze River Delta coastal region is generally in an important window of transition from an industrial to a postindustrial society, where accelerated population mobility further leads to the collapse of traditional ways of interpersonal interaction and modern ways of interpersonal interaction based on the rule of law increasingly become mainstream, in which case the realistic market demand for credit rules gradually increases, and the national model credit policy, through the supply of credit institutions, provides a public market. The National Credit Demonstration Policy (NCP), through the provision of a credit system, effectively curtails the phenomenon of green default from both public and market perspectives, which is a good match for the real needs of cities, and in this case the NCP has a significantly positive impact on the efficiency of the green economy.

### 4.3. National Credit Model Policies and Green Economy Efficiency: Political Variability

Considering the special context of green economic transformation in the Yangtze River Delta and the whole country, different cities’ administrative levels have the potential to influence regional economic and social development to a certain extent. Compared to prefecture-level cities, cities above the subprovincial level (e.g., Shanghai, Nanjing, Hangzhou, Hefei, and Ningbo) have more advantages in terms of project approval, financial support, and resource allocation, but under the mechanism of GDP promotion tournament for local officials, governmental consciousness may have a delaying effect on the mismatch of urban resources and factors and on the deeper reform of the socialist market economy. However, under the mechanism of GDP promotion tournaments for local officials, there may also be a delayed effect of officialism on the maldistribution of resources and factors in cities and the deeper reform of the socialist market economy, which may have a negative effect on the consolidation of the green economic development model and the improvement of green economic efficiency. Therefore, an interaction term between subprovincial and prefectural cities and the national credit demonstration policy was introduced into the benchmark model to discuss the mechanism of the national credit demonstration policy on the green economic efficiency of cities, and the estimated results are compiled in Table 5.

Combining the Log-likelihood, AIC, and SC value magnitudes, models (5) and (9) were selected for the next step of the analysis. The estimation results show that the interaction term x (above subprovincial level, prefecture level) regression coefficient, i.e., the treatment effect coefficient, is 0.042 for cities above the subprovincial level, which passes the 5% significance level test, while the treatment effect is insignificant for prefecture-level cities, indicating significant political variability in the impact of national credit demonstration policies on urban green economic efficiency. The economic explanation is as follows: due to the stage of green development, compared to prefecture-level cities, cities above the subprovincial level have the advantage of concentrating resources such as capital, manpower, and technology under the framework of the national credit demonstration policy to carry out policy refinement and support with a larger scope and wider area, such as improving public credit information systems and building integrated financial service platforms, etc. This reasonable investment of resources can, on the one hand, increase awareness of credit and the rule of law in economic operations. On the other hand, the spillover of the national credit demonstration policy further reduces the endogenous transaction costs in the market and thus improves the level of efficiency of the green economy.

### 4.4. National Credit Demonstration Policies and Green Economy Efficiency: Interprovincial Variability

Under the current institutional mechanism of economic development, interprovincial variability is also an important cause of regional heterogeneity. As an important institutional constraint used to promote the construction of the social credit system, the social credit regulations at the provincial level clarify the powers and responsibilities of all parties in the construction of the social credit system, ensuring that specific credit model policies such as the construction of public credit platforms, the identification and management of red and black lists, the cultivation and development of credit service institutions, and the protection of the rights and interests of credit subjects are implemented on the ground; but, due to interprovincial differences, the social credit regulations of one city and three provinces in the Yangtze River Delta are not synchronised. The Shanghai Social Credit Regulations, Zhejiang Public Credit Information Management Regulations, and Jiangsu Social Credit Regulations came into effect in 2017, 2018, and 2022, respectively, while Anhui only issued the Interim Measures for the Collection and Sharing of Public Credit Information in Anhui Province in 2015, finding that there are obvious differences in the timing and content of credit systems in different provinces of the Yangtze River Delta. These differences potentially play a role in the pathway of national credit model policies, affecting the efficiency of the green economy. Therefore, the interaction term x provincial characteristics were introduced into the benchmark model, and the most appropriate models (OLS, SLM, and/or SEM) were selected for the next step of analysis by combining the Log-likelihood, AIC, and SC value magnitudes; the results are reported in Table 6.

In the case of the interaction term x provincial characteristics alone, the regression coefficient, i.e., the treatment effect coefficient, passed the significance test at the 5% level (coefficient of 0.036) only for Zhejiang Province, while none of the other provinces and cities were significant. In the case of both the interaction term and the interaction term x provincial characteristics, the treatment effect coefficients on the efficiency of the green economy for Shanghai, Jiangsu, and Anhui, with the exception of Zhejiang, passed the test at the 5% level of significance, with decreasing coefficients for Jiangsu, Shanghai, and Anhui, in that order. A possible reason for this is that, as a major area in the development of China’s private economy, SMEs in Zhejiang Province have unique advantages in terms of stimulating market dynamics and absorbing labour, but they also face the challenge of insufficient cross-regional expansion. In comparison with Shanghai, Jiangsu, and Anhui, Zhejiang’s private enterprises’ market operations are mainly local and lack a presence in the Yangtze River Delta region and beyond, which could explain, in terms of the effect of the national credit demonstration policy on the green economy, why the individual effect of Zhejiang is apparent but diluted when included in the analysis of the Yangtze River Delta as a whole. In contrast, the positive effect of the National Credit Demonstration Policy on green economic efficiency is more pronounced in the other provinces when included in the overall Yangtze River Delta analysis framework, despite the less pronounced individual effect. The takeaway from this is that in the context of building a unified national market and constructing a new development pattern of domestic and international dual circulation, it is necessary to give full play to the characteristics of regional advantages and emphasise connotative innovative development on the one hand and to focus on outward synergistic development on the other, so as to allocate resource factors in a larger spatial context and continuously improve the green economic efficiency of the region as a whole.

It is important to note that in traditional econometric teaching, there is a high emphasis on the importance of R-squared to the degree of model fit, arguing that in linear cross-sectional data the R-squared value can reveal how much of the dependent variable can be explained by the independent variable. However, for pragmatic econometricians, the models are much more complex than linear cross-sectional data, and there is no scientific criterion for judging R-squared. However, in Table 3, Table 4, Table 5 and Table 6, we followed the research convention of selecting the significant variables separately for further estimation, and the results show an overall reduction in the R-squared values in the new regressions compared to the original regressions, suggesting that retaining the other control variables provides a better fit to the model. Furthermore, a normal model for empirical analysis will not have all variables pass the significance tests due to multiple factors that may be influenced by the sample distribution, model design, etc. If this were to be achieved, the research design might lose some important information; for example, some variables are important but cannot be eliminated because they are not significant, otherwise the designed model would become less comprehensive. Therefore, in the multiperiod spatial DID model in this paper, we are more concerned with the purpose of the study, the construction of the model, and the significance of the results.

The marginal contributions and innovations of this paper compared with existing studies are: firstly, although some scholars have paid attention to the impact of national policies on green development (e.g., Song et al. used panel data from 308 prefecture-level cities in China from 2003 to 2017 to examine how green industrial policies affect industrial pollution emissions using the same time-varying differential model with the establishment of three national eco-industrial parks as a quasi-natural experiment [28]; Wang et al. explored firms’ green car credit policy in terms of their choice of green technological innovation [29]; and Hong et al. collected panel data from 2007 to 2018 for 2825 listed companies in China based on the “Green Credit Guidelines” released by the China Banking Regulatory Commission in 2012 to investigate the impact of green credit guidelines on corporate green technological innovation and its mechanisms [30]), existing studies either examine the impact of policies on regional green development or the role of credit policies on the green innovation of micro enterprises but do not assess national credit demonstration policies and their impact on green economic efficiency at the regional level, which is the main focus of this paper; secondly, in the process of implementing the national credit demonstration policy, regional heterogeneity is likely to lead to variability in the implementation effects of the policy. Therefore, drawing on Kun Ge et al.’s research ideas [31], the Yangtze River Delta region is chosen as the research object, and cities with different locations and political status and belonging to different provinces are examined in order to better reflect the actual policy. Finally, considering the competing relationships of resource factors in the spatial scale of cities, and based on the Coupling Coordination Model and PVAR Model used by Yi Hu et al. [32], the article adopts a more scientific multi-period spatial DID model to assess the policy effects and impact heterogeneity of national credit demonstration policies on urban green economic efficiency.

## 5. Conclusions

The existing literature examines the effects of national credit demonstration policies, focusing on practice-level analysis but lacks systematic research on the role of green economic efficiency. This paper establishes more reasonable green economy efficiency indicators based on Oh’s research, taking cities above prefecture level in the Yangtze River Delta as the research sample and using a multiperiod spatial DID approach to empirically examine the potential impact of the national credit demonstration policy on urban green economy efficiency and further explores the variability of the impact across different locations, political statuses, and provinces.

The study found the following: (1) National credit demonstration policies have a significantly positive effect on urban green economic efficiency. As an innovative way to build a social credit system, national credit demonstration policies are of great practical significance for strengthening the awareness of market rules and regulations, regulating the market order, and enhancing green economic efficiency. (2) In terms of regional variability, the impact of the national credit demonstration policy on green economic efficiency in coastal cities is significantly positive, but the impact is not prominent in noncoastal cities. Under the new development pattern, the realistic demand for credit regulation in developed, coastal areas has increased, and the national credit demonstration policy has had a catalytic effect on green economic efficiency through matching credit system supply, similar to the economic impact of green credit in the study by Wang et al. [33]. (3) In terms of political variability, the impact of the national credit demonstration policy on green economic efficiency in cities above the subprovincial level is significantly positive, while the effect on green economic efficiency in prefecture-level cities is insignificant. Under the framework of the national credit model policy, cities above the subprovincial level have a greater advantage in terms of policy refinement and support, which reduces market operating costs through policy spillovers and thus improves green economy efficiency, which corresponds to the conclusions drawn by van Langen et al. on the applicability of green deal policy instruments in helping to transform the Dutch circular economy [34]. (4) In terms of interprovincial variability, the impact of the National Credit Demonstration Policy on green economy efficiency in Zhejiang Province is more significant, and the effect is somewhat weakened after being included in the Yangtze River Delta analysis. While the impact of the National Credit Demonstration Policy on the green economic efficiency of other provinces is not prominent, the positive effect of the policy on green economic efficiency is obvious in the framework of the overall analysis of the Yangtze River Delta.

The research implications of the article are as follows: Firstly, the national credit demonstration policy can not only promote the differentiated supply of formal and informal systems in different regions but also become an important exogenous factor influencing the quality of the economy by enhancing the efficiency of the green economy, which provides a new logical starting point for the planning and implementation of pilot demonstration policies with Chinese characteristics and a way of addressing the financing challenges of SMEs under the current environmental regulations in China. Secondly, given the existence of regional economic “lock-in effects,” the impact of national credit demonstration policies on the efficiency of the green economy varies between regions at different stages of development, and therefore differentiated credit measures should be implemented according to regional heterogeneity in order to better achieve regional economic development goals. Again, for cities of different sizes, the rational and regulated use of administrative resources and the reduction of undue administrative intervention in the market allocation of resource factors are both basic requirements for integrated regional development and important prerequisites for the construction of a large national unified market and the enhancement of China’s green economy efficiency. Finally, in the absence of national social credit regulations in the short term, strengthening interprovincial coordination to achieve as much parallel implementation of regional credit policies as possible is conducive to weakening the impact of administrative fragmentation on the effectiveness of credit policy implementation and promoting the effective unification of connotative innovative development and outward synergistic development, which is beneficial to enhancing the overall green economic efficiency of the region, thus realising China’s profound economic transformation.

## Figures and Tables

**Table 1:** Analysis of cities and characteristics in the Yangtze River Delta region.

City	Is It Coastal?	Is it Above the Subprovincial Level?	Province	City	Is It Coastal?	Is It Above the Subprovincial Level?	Province
Shanghai	Yes	Yes	Shanghai	Quzhou	No	No	Zhejiang
Nanjing	No	Yes	Jiangsu	Zhoushan	Yes	No	Zhejiang
Wuxi	No	No	Jiangsu	Taizhou	Yes	No	Zhejiang
Xuzhou	No	No	Jiangsu	Lishui	No	No	Zhejiang
Changzhou	No	No	Jiangsu	Hefei	No	No	Anhui
Suzhou	No	No	Jiangsu	Huaibei	No	No	Anhui
Nantong	Yes	No	Jiangsu	Bozhou	No	No	Anhui
Lianyungang	Yes	No	Jiangsu	Suzhou	No	No	Anhui
Huai’an	No	No	Jiangsu	Bengbu	No	No	Anhui
Yancheng	Yes	No	Jiangsu	Fuyang	No	No	Anhui
Yangzhou	No	No	Jiangsu	Huainan	No	No	Anhui
Zhenjiang	No	No	Jiangsu	Chuzhou	No	No	Anhui
Taizhou	No	No	Jiangsu	Lu’an	No	No	Anhui
Suqian	No	No	Jiangsu	Ma’anshan	No	No	Anhui
Hangzhou	Yes	Yes	Zhejiang	Wuhu	No	No	Anhui
Ningbo	Yes	Yes	Zhejiang	Xuancheng	No	No	Anhui
Wenzhou	Yes	No	Zhejiang	Tongling	No	No	Anhui
Huzhou	No	No	Zhejiang	Chizhou	No	No	Anhui
Jiaxing	Yes	No	Zhejiang	Anqing	No	No	Anhui
Shaoxing	Yes	No	Zhejiang	Huangshan	No	No	Anhui
Jinhua	No	No	Zhejiang				

**Table 2:** Spatial correlation tests for policy implementation effects: the Moran index.

Measurement Indicators	2015	2016	2019
Interaction items (DID)	−0.1600	−0.1442	−0.1442
Green Economic Efficiency	−0.0627	−0.1061	−0.1511
Interaction term (DID) × Green economic efficiency	0.0998	−0.0548	−0.1222

**Table 3:** National credit demonstration policies and green economy efficiency: benchmark model estimation results.

	(1)	(2)	(3)	(4)	(5)	(6)	(7)	(8)	(9)	(10)	(11)	(12)	(13)
Model Type	OLS	SLM	SEM	OLS	SLM	SEM	OLS	SLM	SEM	OLS	SLM	SEM	SEM
Interaction items (DID)	0.010 (0.775)	0.010 (0.866)	0.020 * (1.733)	0.010 (0.751)	0.010 (0.865)	0.020 (1.678)	0.012 (0.847)	0.011 (0.943)	0.027 ** (2.429)	0.012 (0.830)	0.011 (0.933)	0.026 ** (2.352)	0.015 ** (2.242)
X1	0.014 (0.278)	0.030 (0.620)	0.020 (0.495)	0.014 (0.273)	0.030 (0.622)	0.019 (0.470)	−0.0005 (−0.008)	0.017 (0.318)	−0.017 (−0.407)	−0.007 (−0.112)	0.011 (0.202)	−0.034 (−0.729)	
X2	0.016 (0.405)	−0.003 (−0.086)	0.003 (0.086)	0.016 (0.357)	−0.002 (−0.055)	−0.0005 (−0.016)	0.039 (0.740)	0.018 (0.332)	0.054 (1.277)	0.048 (0.817)	0.026 (0.478)	0.069 (1.495)	
X3	0.154 (1.544)	0.157 * (1.756)	0.183 ** (2.353)	0.154 (1.501)	0.158 * (1.744)	0.176 (2.092)	0.162 (1.565)	0.163 * (1.800)	0.197 ** (2.495)	0.158 (1.502)	0.158 (1.738)	0.185 ** (2.310)	0.158 ** (1.331)
X4	−0.341 (−0.213)	−0.004 (−0.002)	−0.918 (−0.812)	−0.345 (−0.206)	0.021 (0.014)	−0.987 (−0.847)	0.183 (0.101)	0.328 (0.205)	−0.115 (−0.101)	−0.012 (−0.006)	0.042 (0.025)	−0.239 (−0.211)	
X5	0.173 (1.348)	0.135 (1.117)	0.160 (1.615)	0.173 (1.326)	0.135 (1.118)	0.155 (1.540)	0.164 (1.245)	0.135 (1.127)	0.164 * (1.785)	0.166 (1.246)	0.133 (1.114)	0.152 * (1.654)	0.167 ** (1.546)
X6				0.0002 (0.010)	−0.001 (−0.067)	0.003 (0.223)	0.002 (0.137)	0.0008 (0.050)	0.007 (0.568)	0.003 (0.189)	0.002 (0.131)	0.009 (0.699)	
X7							−0.788 (−0.796)	−0.558 (−0.610)	−1.428 ** (−1.964)	−0.889 (−0.856)	−0.669 (−0.725)	−1.659 ** (−2.142)	−1.538 ** (−2.343)
X8										0.0291 (0.377)	0.048 (0.693)	0.041 (0.734)	
W_y/LAMBDA		−0.033 (−1.072)	−0.637 *** (−2.640)		−0.033 (−1.07)	−0.642 (−2.662)		−0.027 (−0.835)	−0.874 *** (−3.926)		−0.033 (−0.997)	−0.900 *** (−4.095)	−0.786 *** (−4.253)
R-squared	0.091	0.115	0.230	0.091	0.115	0.231	0.108	0.123	0.336	0.112	0.134	0.351	0.349
Log-likelihood	89.614	90.172	91.386	89.614	90.174	91.411	90.016	90.361	92.973	90.109	90.600	93.237	93.115
AIC	−165.227	−164.343	−168.772	−163.227	−162.347	−166.821	−162.032	−160.721	−167.947	−160.219	−159.199	−166.475	−168.751
SC	−153.232	−150.634	−156.777	−149.519	−146.925	−153.113	−146.609	−143.585	−152.525	−143.083	−140.35	−149.339	−151.227

Note: Regression coefficients are accompanied by t/z values in parentheses below the regression coefficients, and superscripts ***, **, and * indicate 1%, 5%, and 10% statistical significance, respectively.

**Table 4:** National credit demonstration policies and green economy efficiency: locational variability.

	(1)	(2)	(3)	(4)	(5)	(6)	(7)	(8)	(9)	(10)
Model Type	OLS	OLS	SLM	SLM	SEM	SEM	OLS	SLM	SEM	SEM
Interaction item x (coastal, noncoastal)	0.042 (1.626)		0.039 * (1.732)	0.028 ** (1.325)	0.043 ** (1.968)	0.047 ** (1.668)	3.051 × 10^−5^ (0.002)	0.0008 (0.062)	0.015 (1.131)	
X1	0.005 (0.087)		0.018 (0.352)		0.004 (0.082)		0.008 (0.138)	0.025 (0.462)	−0.026 (−0.520)	
X2	0.0592 (1.045)		0.0419 (0.777)		0.051 (1.079)		0.036 (0.631)	0.015 (0.275)	0.057 (1.161)	
X3	0.229 * (2.022)	0.178 ** (1.354)	0.222 ** (2.271)	0.232 ** (2.215)	0.270*** (2.856)	0.342 *** (2.759)	0.148 (1.383)	0.147 (1.595)	0.155 * (1.733)	0.168 * (1.336)
X4	0.127 (0.069)		0.152 (0.095)		0.007 (0.005)		−0.150 (−0.078)	−0.086 (−0.052)	−0.652 (−0.532)	
X5	0.214 (1.598)		0.185 (1.523)		0.189 * (1.846)	0.192 * (1.745)	0.146 (1.103)	0.113 (0.951)	0.119 (1.222)	
X6	0.001 (0.079)		0.0006 (0.039)		0.007 (0.474)		0.005 (0.307)	0.004 (0.256)	0.012 (0.863)	
X7	−1.144 (−1.116)		−0.95 (−1.039)		−1.402 * (−1.717)	−1.301 * (−1.128)	−0.780 (−0.749)	−0.557 (−0.603)	−1.463 * (−1.771)	−1.568 ** (−1.752)
X8	0.003 (0.044)		0.019 (0.275)		−0.006 (−0.084)		0.030 (0.382)	0.050 (0.710)	0.068 (1.109)	
W_y/ LAMBDA			−0.025 (−0.753)		−0.606 ** (−2.491)	−0.634 ** (−1.417)		−0.035 (−1.022)	−0.758 *** (−3.251)	−0.649 *** (−2.216)
R-squared	0.164	0.153	0.175	0.164	0.286	0.278	0.093	0.115	0.266	0.259
Log-likelihood	91.337	91.216	91.617	90.324	93.091	92.913	89.658	90.170	91.694	91.688
AIC	−162.674	−164.542	−161.234	−166.621	−166.182	−168.231	−159.317	−158.34	−163.388	−164.532
SC	−145.538	−149.383	−142.385	−144.873	−149.046	−160.876	−142.181	−139.491	−146.252	−151.343

Note: Regression coefficients are accompanied by t/z values in parentheses below the regression coefficients, and superscripts ***, **, and * indicate 1%, 5%, and 10% statistical significance, respectively.

**Table 5:** National credit demonstration policies and green economy efficiency: political variability.

	(1)	(2)	(3)	(4)	(5)	(6)	(7)	(8)	(9)	(10)
Model Type	OLS	OLS	SLM	SLM	SEM	SEM	OLS	SLM	SEM	SEM
Interaction item x (above subprovincial level, prefecture level)	0.036 (1.537)		0.040 ** (1.979)	0.041 ** (1.996)	0.042 ** (2.164)	0.032 *** (2.667)	−0.0007 (−0.039)	−0.003 (−0.187)	0.013 (0.885)	
X1	0.007 (0.131)		0.029 (0.591)		−0.008 (−0.162)		0.009 (0.150)	0.030 (0.531)	−0.019 (−0.377)	
X2	0.054 (0.956)		0.028 (0.543)		0.066 (1.400)		0.036 (0.622)	0.013 (0.232)	0.049 (1.005)	
X3	0.212 * (1.923)	0.342 ** (1.824)	0.218 ** (2.323)	0.165 ** (2.543)	0.244 *** (2.835)	0.324 *** (2.556)	0.149 (1.390)	0.150 (1.633)	0.167 * (1.900)	0.158 * (1.879)
X4	−0.245 (−0.132)		−0.179 (−0.114)		−0.568 (−0.473)		−0.160 (−0.083)	−0.129 (−0.078)	−0.470 (−0.367)	
X5	0.185 (1.420)		0.147 (1.281)		0.155 (1.626)		0.146 (1.096)	0.110 (0.923)	0.129 (1.304)	
X6	−0.011 (−0.553)		−0.015 (−0.849)		−0.010 (−0.596)		0.005 (0.288)	0.003 (0.202)	0.017 (1.128)	
X7	−0.852 (−0.847)		−0.577 (−0.653)		−1.461 * (−1.832)	−1.415 * (−1.367)	−0.775 (−0.739)	−0.531 (−0.568)	−1.394 * (−1.665)	−1.495 ** (−1.564)
X8	0.033 (0.433)		0.057 (0.858)		0.045 (0.762)		0.030 (0.385)	0.050 (0.719)	0.048 (0.787)	
W_y/ LAMBDA			−0.044 (−1.337)		−0.713 *** (−3.015)	−0.624 *** (−2.014)		−0.035 (−1.034)	−0.694 *** (−2.916)	−0.558 *** (−2.815)
R-squared	0.157	0.148	0.192	0.189	0.314	0.303	0.093	0.116	0.247	0.236
Log-likelihood	91.163	91.123	92.035	91.887	93.361	93.245	89.659	90.186	91.559	91.448
AIC	−162.326	−161.332	−162.069	−163.872	−166.721	−167.342	−159.319	−158.371	−163.118	−164.223
SC	−145.191	−146.761	−143.22	−144.234	−149.585	−159.342	−142.183	−139.522	−145.982	−146.897

Note: Regression coefficients are accompanied by t/z values in parentheses below the regression coefficients, and superscripts ***, **, and * indicate 1%, 5%, and 10% statistical significance, respectively.

**Table 6:** National credit demonstration policies and green economy efficiency: interprovincial variability.

Region	Shanghai			Jiangsu			Zhejiang			Anhui		
	(1)	(2)	(3)	(4)	(5)	(6)	(7)	(8)	(9)	(10)	(11)	(12)
Interaction items		0.029 ** (2.463)	0.027 ** (2.354)		0.032 ** (2.523)	0.035 ** (2.256)		0.017 (1.312)			0.028 ** (2.034)	0.047 ** (2.643)
Interaction items × provincial characteristics	0.0004 (0.013)	0.022 (0.661)		0.001 (0.069)	−0.019 (−1.004)		0.036 (2.124)	0.02 (1.199)		0.022 (0.965)	−0.006 (−0.200)	
X1	−0.004 (−0.085)	−0.044 (−0.898)		−0.005 (−0.103)	−0.021 (−0.436)		−0.024 (−0.507)	−0.034 (−0.727)		0.003 (0.061)	−0.038 (−0.756)	
X2	0.042 (0.857)	0.073 (1.577)		0.042 (0.861)	0.063 (1.376)		0.059 (1.267)	0.068 (1.492)		0.043 (0.887)	0.070 (1.513)	
X3	0.181 * (1.907)	0.208 ** (2.396)	0.212 ** (2.497)	0.181 (2.068)	0.191 ** (2.399)	0.123 *** (2.965)	0.256 *** (2.920)	0.233 *** (2.635)	0.245 *** (2.687)	0.148 (1.591)	0.195 ** (2.084)	0.356 ** (2.439)
X4	−0.619 (−0.458)	−0.334 (−0.295)		−0.636 (−0.470)	0.219 (0.180)		0.347 (0.271)	0.295 (0.245)		−0.513 (−0.397)	−0.248 (−0.219)	
X5	0.125 (1.159)	0.137 (1.455)		0.125 (1.227)	0.153 * (1.677)	0.678 * (1.753)	0.181 * (1.848)	0.180 * (1.926)	0.107 * (1.967)	0.107 (1.060)	0.158 (1.631)	
X6	0.011 (0.804)	0.008 (0.591)		0.012 (0.814)	0.005 (0.404)		0.011 (0.847)	0.009 (0.726)		0.006 (0.419)	0.010 (0.723)	
X7	−1.245 (−1.393)	−1.857 ** (−2.264)	−1.556 ** (−2.245)	−1.246 (−1.460)	−1.638 ** (−2.131)	−1.429 ** (−2.143)	−1.456 * (−1.834)	−1.600 ** (−2.094)	−1.769 ** (−2.876)	−1.371 (−1.628)	−1.651 ** (−2.132)	−1.543 ** (−2.862)
X8	0.0490 (0.775)	0.039 (0.688)		0.051 (0.755)	0.005 (0.079)		−0.005 (−0.0761)	0.004 (0.061)		0.040 (0.636)	0.043 (0.758)	
W_y/LAMBDA	−0.578 ** (−2.363)	−0.91 *** (−4.168)	−0.989 *** (−3.337)	−0.583 (−2.387)	−0.878 *** (−3.955)	−0.865 *** (−3.532)	−0.757 *** (−3.244)	−0.889 *** (−4.024)	−0.874 *** (−3.642)	−0.640 (−2.651)	−0.904 *** (−4.120)	−0.766 *** (−4.874)
Model type	SEM	SEM	SEM	SEM	SEM	SEM	SEM	SEM	SEM	SEM	SEM	SEM
R-squared	0.213	0.361	0.349	0.214	0.362	0.351	0.318	0.371	0.369	0.241	0.353	0.346
Log-likelihood	91.244	93.454	92.542	91.246	93.733	93,499	93.207	93.943	93.876	91.684	93.257	93.123
AIC	−162.488	−164.908	−165.459	−162.492	−165.466	−166.342	−166.414	−165.886	−166.324	−163.368	−164.515	−164.652
SC	−145.352	−146.058	−147.112	−145.357	−146.617	−147.325	−149.278	−147.037	−148.235	−146.232	−145.665	−46.312

Note: Regression coefficients are accompanied by t/z values in parentheses below the regression coefficients, and superscripts ***, **, and * indicate 1%, 5%, and 10% statistical significance, respectively.

## Data Availability

All data generated or analysed during this study are included in this published article.
